# A hybrid technique combining intramedullary pinning with extramedullary plate fixation in unstable and comminuted radial head fractures following on-table reconstruction

**DOI:** 10.1186/s12891-021-04498-w

**Published:** 2021-07-09

**Authors:** Xu Gao, Shi-you Dai, Hai-lei Yin, Fei Li, Yong-qiang Sui, Rui Huang, Hai-yu Fan

**Affiliations:** 1 Department of Orthopaedic Surgery, Qingdao University, Qingdao City, 266071 P.R. China; 2 Department of Bone, Joint and Sports Medicine, East District, Qingdao Municipal Hospital, Qingdao City, 266071 P.R. China; 3 Department of Second Orthopaedic Surgery, No. 971 Hospital of the People’s Liberation Army (PLA), Qingdao City, 266071 P.R. China; 4 Department of State Key Laboratory for Marine Corrosion and Protection, Luoyang Ship Material Research Institute, Qingdao City, 266071 P.R. China; 5 Department of Burn and Plastic Surgery, No. 971 Hospital of the People’s Liberation Army (PLA), Qingdao City, 266071 P.R. China

**Keywords:** Radial head, Comminuted fracture, Intramedullary pinning, On-table reconstruction technique

## Abstract

**Background:**

Management of comminuted radial fractures remains controversial. Currently, the emergence of on-table reconstruction technique has made fixation in comminuted radial head fractures more viable. However, the present study reported an intro-operative unstable displacement from the reconstructed radial head to the neck during plate fixation, characterized by a poor radiocapitellar contact and incongruity between the radial head and neck. A hybrid technique combining with intramedullary pining was performed in our study to restore the normal alignment and maintain the stability of fixation. Therefore, the purpose of this article aimed to prove the feasibility of unstable comminuted radial head fractures treated with the extramedullary plate and intramedullary pinning fixation using titanium elastic nails.

**Methods:**

The clinical, functional and radiographic outcomes of the groups were compared during follow-up. The radiographic examination was conducted to evaluate the status of bone union, heterotopic ossification and post-traumatic arthritis. The functional assessment was performed to evaluate clinical effects, which included measurements of range of motion (ROM) in the elbow, Visual Analog Scale (VAS) score, Elbow Self-Assessment score (ESAS), Mayo Elbow Performance score (MEPS), and Disabilities of the Arm, Shoulder, and Hand (DASH)Outcome Measure score.

**Results:**

Thirteen patients with unstable fractures were participated with an average follow-up of (38.6 ± 4.5) months for the experimental group and (32.0 ± 6.3) months for the control group, respectively. The functional outcomes in the experimental group, including MEPS and DASH, were significantly superior to the control group. However, no significant difference was observed in the elbow ROM and VAS score between two groups. In the last follow-up, one patient with post-traumatic arthritis rated as grades 1 and two with heterotopic ossifications were observed in the experimental group. In the control group, degenerative changes were observed in three cases (grade 1 in two cases and grade 2 in one case) and heterotopic ossifications rated as grade I were found in two patients.

**Conclusion:**

Collectively, intramedullary pinning with extramedullary plate fixation is feasible in unstable comminuted radial head fractures, which can be considered as a remedial surgery for on-table reconstruction technique.

## Background

Mason type III fractures refer to comminuted fractures involving the entire radial head displaced from the shaft [1]. The common surgical treatments for such complicated injuries, such as radial head resection (RHR), radial head arthroplasty (RHA) and open reduction and internal fixation (ORIF), have their merits and demerits, respectively. Since there is lack of long-term and large-volume studies in comminuted radial head fractures, the optimal treatment has not reached a consensus [2, 3]. Currently, it has been well recognized that the radial head is an important stabilizer of the forearm and elbow [4]. The emergence of specific radial head implants and innovative techniques have made ORIF preferable to preserve the integrity of the fractured radial head [5, 6].

On-table reconstruction technique is first described by Businger et al., which allows the radial head fragments fixed on the operation table with preliminary fixation using reduction clamps and 1 mm Kirschner wires [7]. This technique provides an optional treatment method and solves a critical challenge that ORIF is almost impossible to repair comminuted fractures with more fragments. Subsequent published series have proved the availability of this technique. Kiran et al. [8] have shown that following Ex situ reconstruction technique, six patients with Mason type-III radial head fractures have a satisfactory motion of elbow with the mean Broberg and Morrey score of 90 points. But non-union occurs in three patients and avascular necrosis in one patient in their study. Latterly, in another study by Everding et al. [9], the mean Mayo Elbow Performance score is 82 points and DASH score is 20 points with asymptomatic non-union occurred in one patient, and no signs of avascular necrosis are observed. Additionally, several on-table techniques using polylactide pins or surgical glue are reported with satisfactory to good surgical outcomes [10, 11]. Accordingly, this technique provides an optional solution for complicated radial head fractures. Nevertheless, since only a few studies have investigated small amounts of patients with conflicting complication rates, the treatment effects of on-table technique may be discrepant in different cases and its reliability needs to be evaluated cautiously in the practical surgical process.

In the present study, we found that some reconstructed radial heads after mini T-type plate fixation were prone to displace from the radial neck during surgery, characterized by a poor radiocapitellar contact and incongruity between the radial head and neck, and we named it unstable comminuted radial head fractures. In these cases, RHR may be considered as a remedial solution since internal implant has largely lost its effects. However, in the present study, a hybrid technique that combined intramedullary pinning with extramedullary plate fixation was manipulated to maintain the stability of fixation and restore the normal alignment in these unstable fractures, with RHR avoided. Additionally, we also retrospectively compared a series of patients surgically treated with RHR or our hybrid technique due to unstable comminuted radial head fractures, and to further assess the clinical and radiological outcomes of this technique. Therefore, the purpose of this article mainly was to ascertain whether the intramedullary pining fixation could be a remedial operation for unstable comminuted radial head fractures after on-table reconstruction technique.

## Methods

### Patients

Between January 2012 and February 2019, a total of 62 consecutive patients with comminuted radial head fractures were reviewed, retrospectively. The primary inclusion criteria were set as follows: (i) Fractures involved entire radial head with three or more fragments, and the radial head was completely displaced from the shaft of radius (Mason type-III); (ii) definitive treatment consisted of RHR or ORIF by extramedullary plate and intramedullary pinning fixation using titanium elastic nails (TEN); and (iii) the follow-up time should be no less than 12 months. The exclusion criteria were previous mobility dysfunction of the injured limb, osseous maturity with closed epiphysis, and the injured limb with fractures of other parts of forearm.

The choice of treatment for type-III fracture was made preoperatively or intraoperatively. We preferred an approach that can preserve the integrity of the radial head in most cases. Hence most patients were initially accepted open reduction and internal fixation to evaluate the feasibility of preserving the radial head. Since the RHR was mostly chosen to fix the fractures that metallic implants couldn’t manage, so the severity of fracture of patients who accepted RHR mostly were much worse than patients who received plates combining intramedullary pinning fixation. The lack of consistency between patients and the influence of selection bias made it difficult to compare the efficiency of RHR with plates combining intramedullary pinning fixation. To minimize the impact of differences between patients, we further made the criteria of inclusion to find the intersection. Basis of principles to choose patients who accepted RHR included: (i) the radial head fracture with partial fragments broken and failed to perform the “on-table” reconstructive operation during surgery; (ii) the integrity of reconstructed radial head couldn’t be maintained stably; (iii) and the reconstructed radial head was not able to bear the axial support provided by the intramedullary pinning and had a risk of increasing fracture displacement.

The diagnosis was confirmed by the computed tomography (CT), anteroposterior and lateral fluoroscopic views of the elbow and wrist, so as to identify other concomitant injuries. Based on the above-mentioned inclusion and exclusion criteria*,* one case of mason type-III fracture with *posterolateral rotational instability injury of the elbow joint (PLRI) *[12] and one patient with a *Monteggia lesion (Jupiter type 2a) *[13] was excluded, respectively. Consequently, a total of 13 mason type-III radial head fractures comprised of 8 males and 5 females were identified at our institution. According to different surgical solutions, six patients treated with ORIF by extramedullary plate and intramedullary pinning fixation were defined as the experimental group, with seven patients who underwent RHR as the control group. The demographic data was shown in Table 1. Four cases were caused by falling during sports activities, and nine cases of injuries were attributed to traffic accidents. The experimental group consisted of 3 males and 3 females with a mean age of (37.1 ± 9.0) years, and the control group included 5 males and 2 females with a mean age of (41.7 ± 7.7) years. There was no significant difference in age between experimental group and control group (*P* = 0.712).

**Table 1:** Details of patient demographics

	ORIF (experimental)	RHR (control)
Patients [13]	6	7
Male [8]	3	5
Female [5]	3	2
Mean ± SD age [years]	37.1 ± 9.0	41.7 ± 7.7
Side of the injury [13]	-	-
Right [9]	4	5
Left [4]	2	2
Mean ± SD time of follow-up [months]	*3*8*.*6 ± 4*.*5	32.0 ± 6*.*3
Mode of injury [13]	-	-
Falling injury [4]	3	1
Traffic accident [9]	3	6

### Surgical technique

**Fig. 1:**
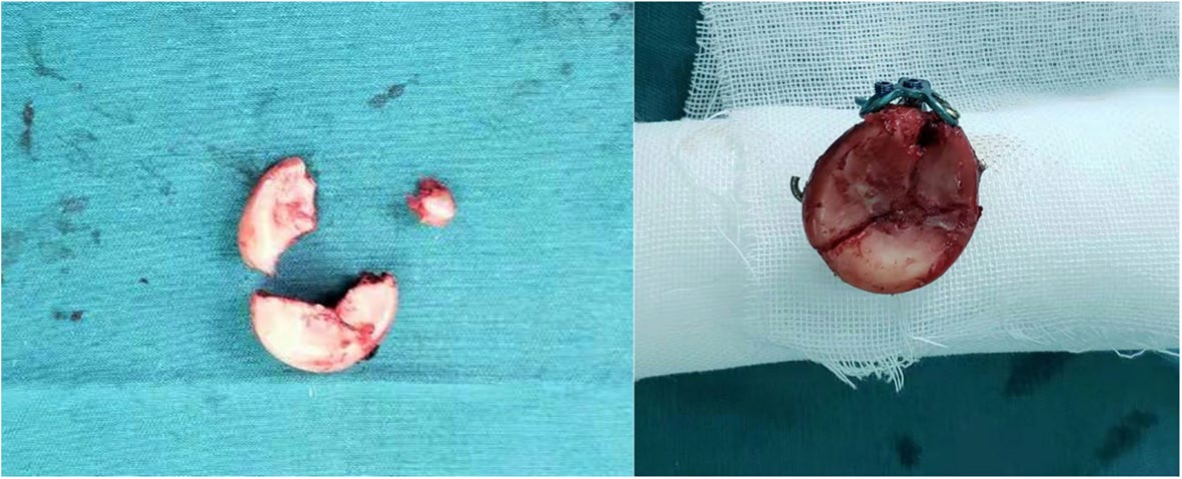
The radial head fragments were retrieved from the joint and reconstructed using k-wires and locking screws of the mini T-type plate on the operation table

**Fig. 2:**
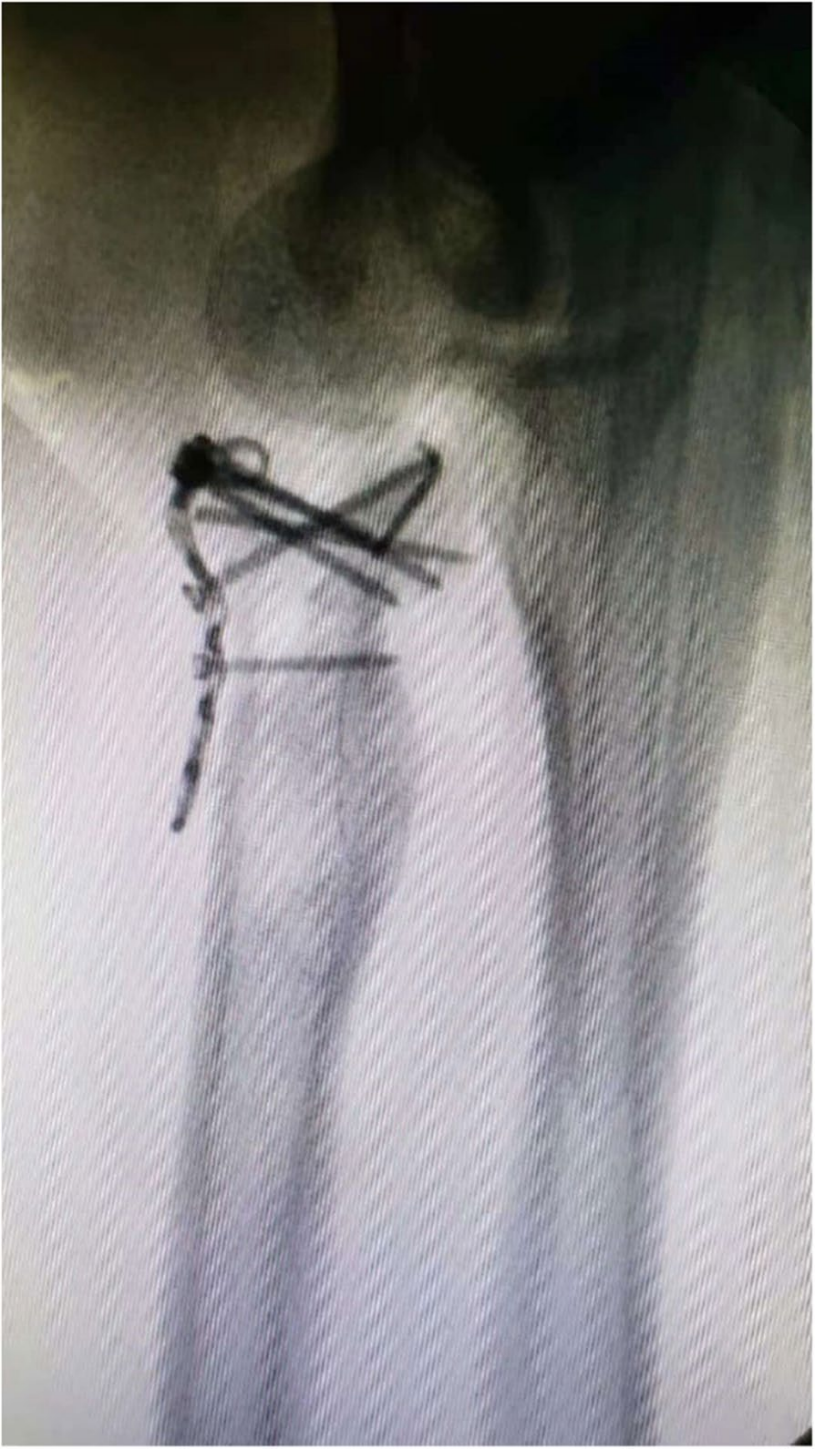
The radial head was replanted into the joint and fixed to the radial shaft

**Fig. 3:**
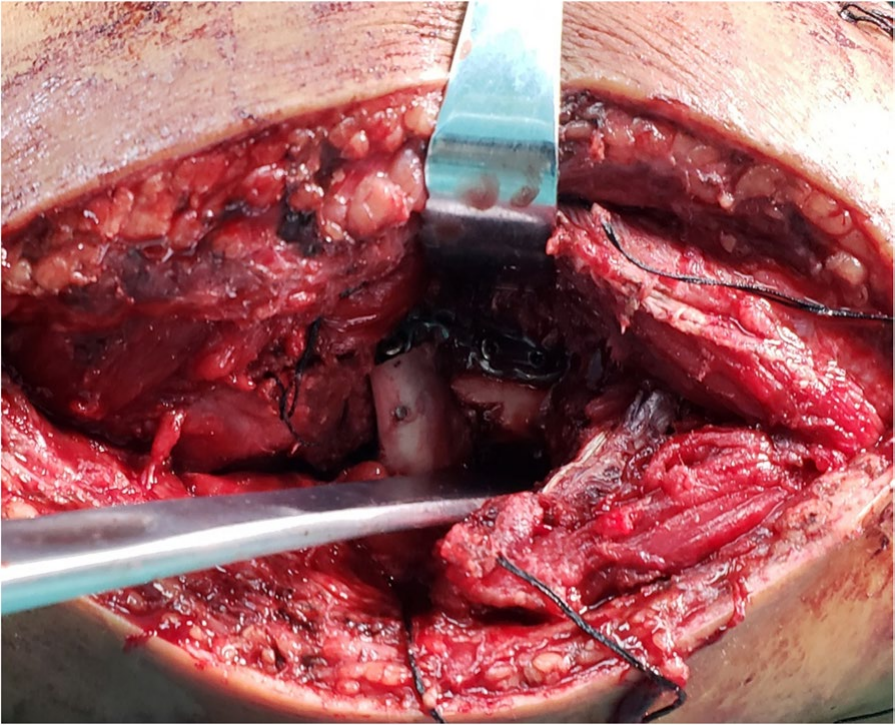
Displacement of the radial head from the neck with a poor alignment under direct vision

All patients were placed in the supine position under general anesthesia, and a lateral Kocher approach was selected to access the radial head. Subsequently, the radial head fragments were retrieved from the joint on the operation table. With the radial head reconstructed using k-wires, two or three 1.5 mm locking screws were inserted through the proximal holes of the mini T-type plate (DOUBLE, mini metal locking plate system, DOUBLE MEDICAL, China) and integrated the plate with the reconstructed radial head (Fig. 1). Based on comparing the fracture line at the radial head base with the corresponding line at the radial neck, the radial head was replanted into the joint and fixed to the radial shaft with two to three screws through the distal holes, which was placed on the correct lateral position to avoid interfering elbow rotation (Fig. 2). With the radial head fixed, stability of the fixation was checked clinically. Strikingly, displacement and tilt could be observed from the reconstructed radial head to the neck, leading to a poor radiocapitellar relationship and loss of normal alignment (Fig. 3). Insufficient stability in the fracture site was verified by probing the radial head with fingers or vessel forceps.

**Fig. 4:**
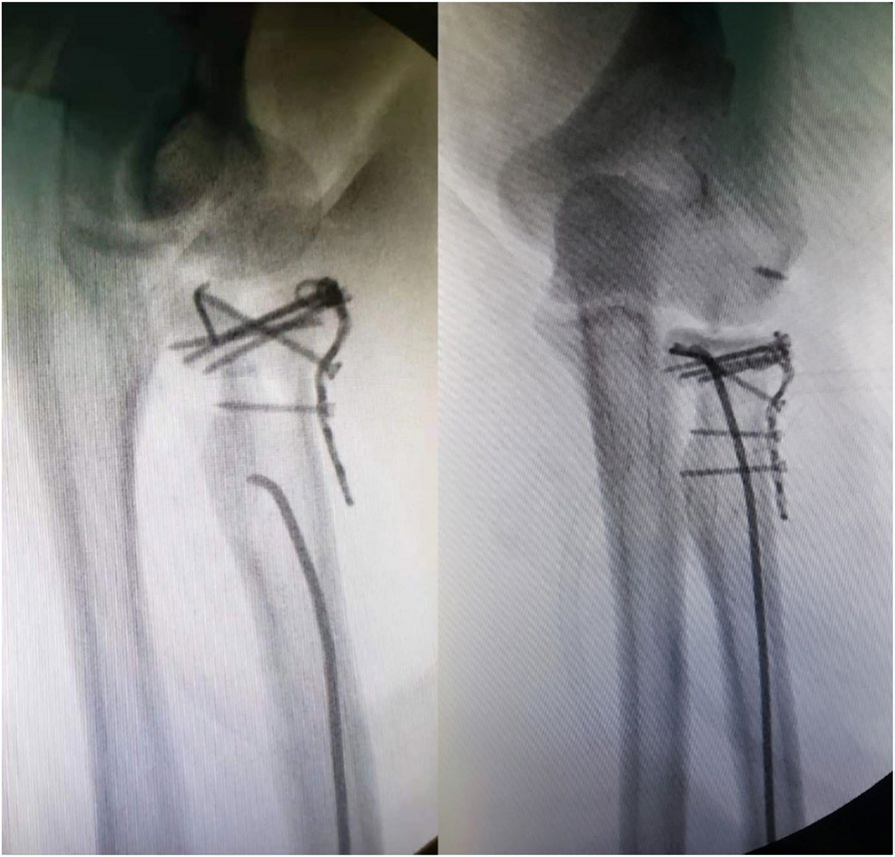
With the TEN upward, the displaced and tilted radial head was gradually repositioned

In the experimental group, patients were treated with intramedullary pining fixation using 2.0 mm TEN (Johnson, titanium elastic nails system, Johnson, America). The proximal position of TEN was bent approximately 25–30 degrees. It then was inserted into the medullary space from the proximal radial styloid process. The TEN was hammered slowly upward until the tip was fixed to the subchondral bone, by which the displaced and tilted radial head was gradually repositioned (Fig. 4). After achieving satisfactory alignment restoration between the radial head and neck under fluoroscopic control, the end of TEN was cut. Conversely, in the control group, the radial head was resected with a bone saw close to the surgical neck. Next, the capsule and annular ligament were sutured with absorbable sutures in both groups, and anchor system was used to repair the avulsion of radial collateral ligament.

### Postoperative rehabilitation

All patients in both two groups were slung for two weeks, followed by early mobilization. Early active-assisted flexion–extension and pronation-supination were commenced from the first postoperative day. The time to allow weight-bearing or other heavy loading activities were based on the clinical and radiological follow-up assessment.

### Evaluation

The follow-up evaluation included functional and radiographic assessment, which was carried out by two orthopedic surgeons who were not involved in the operation. The functional assessment consisted of measurements of range of motion (ROM) in the elbow, Visual Analog Scale score (VAS), Elbow Self-Assessment score (ESAS) [14], Mayo Elbow Performance score (MEPS) [15] and Disabilities of the Arm, Shoulder, and Hand (DASH)Outcome Measure score [16]. The ESAS mainly evaluated the patient’s satisfaction with the elbow using a scale of 1–6: 1, very good; 2, good; 3, satisfied; 4, sufficient; 5, insufficient; and 6, poor.

Postoperative radiographic assessment was based on both the anteroposterior and lateral fluoroscopic views of the elbow to evaluate the status of bone union, heterotopic ossification, joint incongruity, avascular necrosis, and post-traumatic arthritis until the last follow-up. The degree of heterotopic ossification was assessed according to Hastings and Graham system [17]. Radiographic signs of post-traumatic arthritis were stratified by the Broberg and Morrey system [18]:

- a normal joint as grade 0
- slight narrowing joint space with the occurrence of minimum osteophytes as grade 1
- moderate narrowing joint space and osteophyte formation as grade 2
- disappeared joint space as grade 3

### Statistical analysis

The independent samples t-test was conducted using SPSS 20.0 (SPSS Inc, Chicago, IL, USA) to assess the statistical significance of differences between the two groups. A p-value of less than 0.05 was considered significant.

## Result

**Fig. 5:**
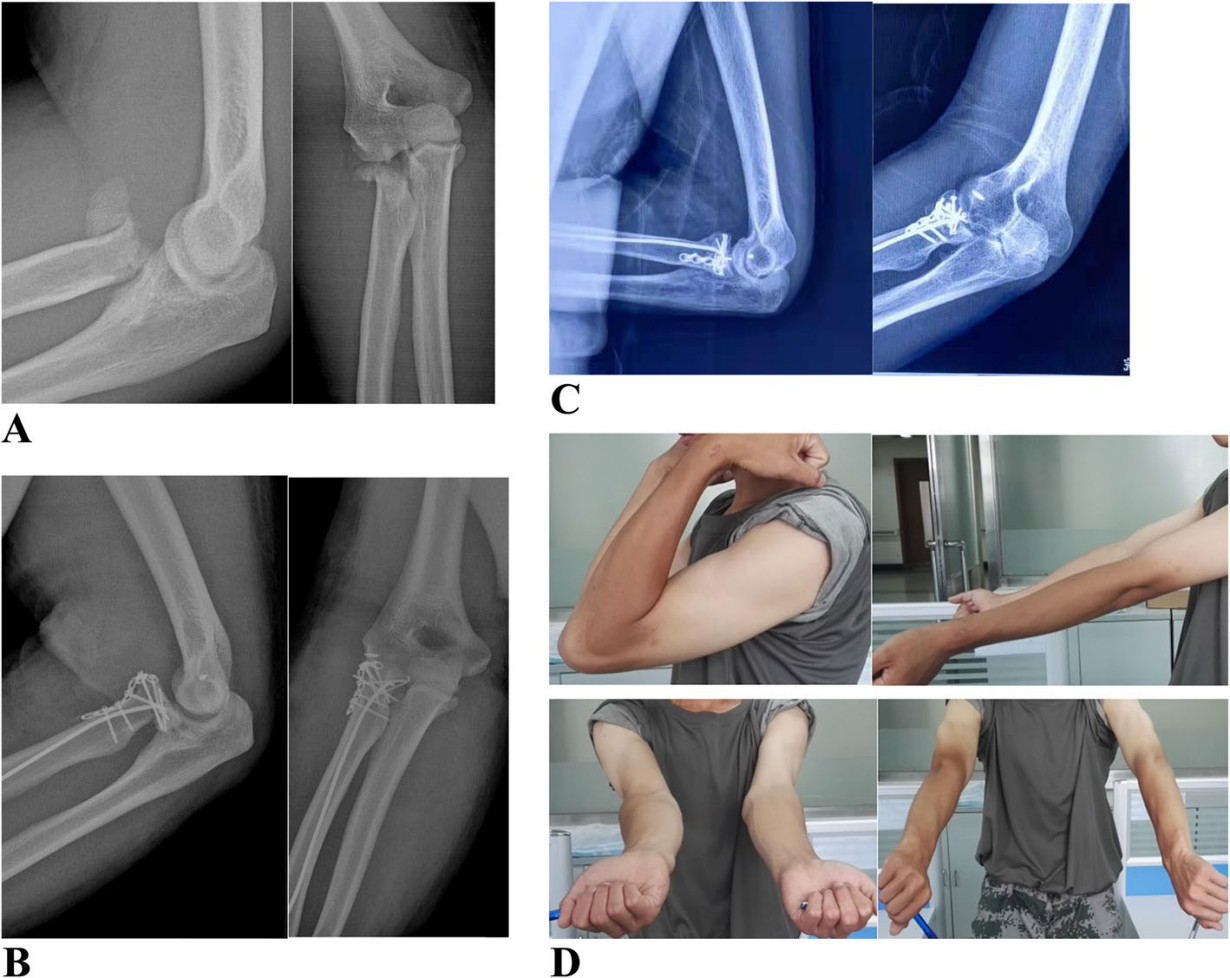
Typical case: left radial head fracture. **a** Preoperative radiograph showed Mason type III radial head fracture. **b** Postoperative radiograph revealed a good restoration of alignment between the radial head and neck. **c** Anteroposterior and lateral radiograph showed bone union at 12 months after surgery. **d** Functional outcomes at the final follow-up. Compared with the unaffected sides, the ROM of elbow and forearm rotation was sufficient

Patients participated in the current analysis with an average follow-up of (38.6 ± 4.5) months for the experimental group and (32.0 ± 6.3) months for the control group, respectively. Figure 5 illustrated the typical cases.

### Intraoperative comparison

The mean operating time for the experimental group was (126.5 ± 9.9) min and (121.0 ± 16.7) min for the control group, which was not significantly different (*p* = 0.136). There was also not significant difference in the mean blood loss during surgery, which was (133.5 ± 13.8) ml to (124.8 ± 14.1) ml for the experimental and control groups, respectively (*p* = 0.920). The fluoroscopy times during surgery differed significantly and was (10.7 ± 3.8) to (3.8 ± 1.6) between the experimental group and control group (*p* = 0.039) (Table 2).

**Table 2:** Intraoperative comparison of the groups

	ORIF (experimental)	RHR (control)	p
Mean ± SD operating time [min]	126.5 ± 9.9	121.0 ± 16.7	0.136
Mean ± SD blood loss [ml]	133.5 ± 13.8	124.8 ± 14.1	0.920
Mean ± SD fluoroscopy times [time]	10.7 ± 3.8	3.8 ± 1.6	0.039

### Functional assessment

The clinical, functional and radiographic outcomes of the groups were summarized in Table 3. At the last follow-up, the mean arc of elbow motion (flexion–extension) in the experimental group was (133.7 ± 8.7) and differed with (130.1 ± 10.2) for the control group, but there was no difference (*p* = 0.856). Additionally, the average pronation is (74.3 ± 7.3) to (75.4 ± 7.4) (*p* = 0.910), and the supination is (68.5 ± 5.5) to (69.0 ± 4.1) (*p* = 0.413), which were also not statistically different. All patients in both two groups could participate in general sports activities without functional limitation of the elbow and joint stiffness.

**Table 3:** The clinical, functional and radiographic outcomes of the groups

	ORIF (experimental)	RHR (control)	p
Mean ± SD VAS	2.3 ± 0.8	3.0 ± 1.3	0.638
Mean ± SD MEPS	87.0 ± 6.7	78.6 ± 2.9	0.016
Mean ± SD DASH	8.7 ± 2.1	17.1 ± 4.1	0.025
Mean ± SD arc of elbow motion	133.7 ± 8.7	130.1 ± 10.2	0.856
Mean ± SD pronation	74.3 ± 7.3	75.4 ± 7.4	0.910
Mean ± SD supination	68.5 ± 5.5	69.0 ± 4.1	0.413
Arthritis [4]	-	-	-
Grade 1 [3]	1	2	-
Grade 2 [1]	0	1	-
Heterotopic ossifications rated as Grade 1 [4]	2	2	-
Elbow Self-Assessment [13]	-	-	-
Very good [1]	1	0	-
Good [5]	2	3	-
Satisfied [5]	3	2	-
Sufficient [2]	0	2	-

*VAS* Visual Analogue Scale, *MEPS* Mayo Elbow Performance Score, *DASH* Disabilities of the arm, shoulder and hand

According to the ESAS, 1 case of very good, 2 cases of good, 3 cases of satisfied were rated in the experimental group. In the control group, 3 patients were rated good, 2 satisfied, and 2 sufficient.

The mean VAS score for pain was (2.3 ± 0.8) for the experimental group and (3.0 ± 1.3) for the control group (*p* = 0.638), respectively, which was not significantly different. Two patients in the experimental group and four in the control group complained of mild pain in the elbow when the weather changes or the injured arm exercises strenuously. However, none of patients considered that the pain affected the daily activities.

Based on the criteria of the MEPS, the mean score of MEPS was (87.0 ± 6.7) to (78.6 ± 2.9) between the experimental group and control group at the last follow-up (*p* = 0.016). The mean DASH score was (8.7 ± 2.1) for the experimental group and (17.1 ± 4.1) for the control group (*p* = 0.025), respectively. Functional changes of the elbow in two groups were significantly different.

### Radiographic outcomes

In the experimental group, all reconstructed radial heads survived without signs of avascular necrosis at the final follow-up. Four patients obtained bone union at three months after surgery, with a normal alignment between the axis of radial head and shaft of radius. Two patients appeared as a delayed union and eventually healed six to eight months postoperatively. Loss of reduction or failure in fixation was not observed. Mild signs of post-traumatic arthritis (grades 1) were observed in one patient, and heterotopic ossifications rated as grade I were found in two patients. In the control group, degenerative changes in elbow rated as grade 1 were observed in two patients and grade 2 in one patient. Additionally, heterotopic ossifications rated as grade I were found in two patients.

## Discussion

At present, there is considerable conflicting evidence in the literature regarding the treatment of comminuted and displaced fractures of the radial head [2, 3, 6]. RHR as early treatment was once considered as a simple and useful approach. Clinical and radiological results from a retrospective review have documented good recovery in MEPS (from 79 to 100), DASH (from 4 to 15), and an acceptable elbow ROM (average 120°)following radial head resection [19]. Mebouinz et al. [20] have demonstrated radial head resection can restore elbow functionality at a rate of 81%. However, due to the damaged radiocapitellar contact, RHR has an increased risk of complications such as instability of valgus or posterolateral rotation, decreased strength, and degenerative changes in elbow and wrist [21]. Chen et al. [22] have reported that relative to RHR, RHA and ORIF can maintain higher Broberg-Morrey elbow joint scores, and they both show better efficacy in reducing pain, larger range of motion, and lower complication rates. Also similar to our study, seven patients with ORIF had a higher elbow functional scores compared with control group. The results were accordant with previous views that ORIF was a good option for Mason type III fractures with a low level of associated injury [23]. Nevertheless, no significant difference in the mean values of ROM of the injured elbow between two groups was found in the present study.

Biomechanical researches have proved the critical role of the radial head as a secondary stabilizer against both valgus and posterolateral instability of the elbow, especially in those cases with the medial collateral ligament or interosseous membrane of the forearm injury [4, 24]. Thus, being aware of the importance of the radial head for elbow function, RHA and ORIF are preferable in restoring normal radiocapitellar contact and anatomical structure. Several excellent short to midterm results have shown better operation outcomes in comminuted radial head fractures treated with RHA, which might be a preferred solution for such fractures but have inherent drawbacks, such as heterotopic ossification, joint stiffness, hardware loosening, and radiocapitellar osteoarthritis, particularly when the prosthesis overstuffs the radiocapitellar joint and further alters the biomechanics of the radial head [25–27]. Consequently, ORIF might be the initial surgical solution to retain the integrity of the fractured radial head, so as to maintain normal articulation and bone stock. If ORIF fails early, it can still be converted into RHA.

A majority of previous studies have reported that comminuted radial head fractures with operative fixation have a high failure rate, nonunion, and osteonecrosis [28]. Moreover, severe Mason type-III radial head fractures are usually defined as “unsalvageable” or “unrepairable” type, which appears impossible to be treated with ORIF [29]. Nevertheless, the progress in implant technology and new fixation techniques have gradually overturned the established views. Particularly, on-table reconstruction technique has been reported and verified clinically. Several studies that exclusively focus on such technique for the complex radial head fractures have revealed satisfactory functional outcomes [7–9]. This technique is not limited to the narrowing anatomical joint space, and allows the reconstructed radial head to replant with the use of plate, which should be worthy of supporting and popularizing. However, in view of the different characteristics of comminuted radial head fractures, difficult extent to fix fragments and a variety of internal implants, the stability of fixation in the reconstructed radial head tends to be discrepant and remains to be assessed in different cases.

Koslowsky et al. [30] have compared four different fixation techniques for the reconstruction of Mason type III fractures in the sawbones model, and find that displacement of the radial head from the neck often occur during plate fixation. Similarly, this issue was observed in the present study after on-table reconstruction of the radial head, leading to a poor radiocapitellar contact and incongruity between the radial head and neck. It is well known that treatment of radial head fractures mainly aims to maximize elbow movement and stability to maintain function [31]. Correct alignment of the radial neck to the reconstructed radial head is crucial for normal rotation in the proximal radioulnar joint. Therefore, only reconstruction of the radial head fails to meet the treatment criterion. Furthermore, many biomechanical studies have shown that the stability in the coronal and sagittal plane largely depends on the anatomical restoration of the radiocapitellar relationship [32].

We analyzed that the unstable displacement might be correlated to the insufficient axial support provided by plate fixation. Both unfirm anchoring force and weak supporting strength from proximal screws to the reconstructed radial head were critical factors. Primarily, the reconstructed radial head with Kirschner wires might block inserting of the proximal screws, and the plate must be placed at “safe-zone,” which mainly limited the amounts, location, and direction of the screws. Moreover, the shape of fracture fragments was not uniform in different cases, so that the expected effects of manipulating on-table reconstruction remained uncertainties. The power drill, Kirschner wires, and proximal screws all probably would break the fragments and made reduction and fixation more complicated. Additionally, differences in plate type and operator’s surgical experience could affect the definitive outcomes, which might be inconsistent with previously reported results. Conclusively, all above-mentioned factors might influence the anchoring force and supporting strength of screws, causing instability between the radial head and neck during plate fixation.

If plate fixation fails to offer reliable axial support, a hybrid technique combining with intramedullary pinning seems useful to compensate for that deficiency. The intramedullary pinning technique is always manipulated in children radial neck fractures, which allows minimally invasive and is easily performed, representing a favorable result with few complications [33]. Sandmann et al. [34] first report that intramedullary pinning not only suits to radial neck fractures in children but satisfactory in adults. A series of superior results have confirmed the feasibility of adult radial neck fractures fixed with intramedullary pinning. Gao et al. [35] has conceived a hypothesis that radial neck component could be stabilized by intramedullary pinning fixation following open reduction and plates or screws fixation in radial head fractures. This hybrid technique was manipulated and verified in our study, by which an additional axial support was obtained in the medullary cavity to prevent the displacement of reconstructed radial head from neck, and maintain restoration of radiocapitellar contact. Also, the use of elastic nail was modified in the present study. The proximal part of the elastic nail was bent, and formed three-point supporting consisting of the nail tip, bending point and nail bottom, which further enhance the axial support to avoid longitudinal displacement of the radial head.

Early failure in plate fixation is not infrequent, particularly in severe comminuted fractures. Ring et al. [28] have shown that 3 of 14 fractures (21.4%) with more than three articular fragments have failure in plate fixation within the first month. Therefore, if there is lack of stability and loss of normal alignment between the reconstructed radial head and neck during plate fixation, incorporating the use of intramedullary pining appears applicable. Moreover, stable fixation in fracture site allows patient to functional exercise early and promotes bone union. Kiran Kumar et al. [8] have reported that only 3 of the 6 patients treated with on-table reconstruction achieve fracture healing after a mean follow-up of 25 months, and one case occurs avascular necrosis of radial head. There is a strong possibility that these complications are attributed to the unstable plate fixation. Comparatively, in the present study, seven patients following additional intramedullary pinning fixation had obtained bone union without avascular necrosis of radial head observed during follow-up. This hybrid technique preserved the integrity of radial head and provided satisfactory elbow function compared with RHR, which was available for selection following unstable plate fixation.

Objectively speaking, there are still some risks and limitations to this technique. Firstly, after plate fixation, distal screws may block insertion of elastic nail. Actually, the axial direction of nail tip is designed to have an angle of approximately 30 degrees with nail shaft. During elastic nail insertion, we suggest that the direction in which nail tip point on the coronal plane should try to keep parallel with the distal screws, and in this way that nail tip can pass through distal screws with the minimum contact area. Additionally, the width of the proximal radial medullary cavity is about 6–7 mm, and the diameter of distal screws we used is 1.5 mm, thus there is a sufficient interspace between screws and medial cortical bone to allow 2.0 mm-sized elastic nail insertion. Secondly, inserting the elastic nail may penetrate the articular surface through the inter-fragmental gap, and cause failure in intramedullary fixation. In addition, the nail tip might potentially lift the fracture fragments, with the articular surface secondary displaced. Therefore, this technique needs the accumulation of operating experiences and learning curves, and the axial support provided by the elastic nail requires further biomechanical exploration. We suggest using the intraoperative fluoroscopy to control the inserting depth of nail, till the nail tip has advanced to the subchondral bone. Lastly, we note that the small number of cases, duration of follow-up and other potential factors could affect the evaluation of the two treatment methods. The influence of selection bias of limited cases may also have affected the evaluation of the results of experimental group in the present study. Hence it needs more quantitative analysis, multi-center studies and patient accumulation to assess the clinical effects of this technique.

## Conclusion

On-table technique can offer an optional solution in the surgical treatment of comminuted radial head fractures. However, if there is unstable displacement from the reconstructed radial head to the neck during plate fixation, intramedullary pinning technique can be selected and applied as a remedial option. Indeed, this hybrid approach availably decrease the risks of failure in fixation and provide a good clinical result.

## Data Availability

The datasets used and/or analysed during the current study are available from the corresponding author on reasonable request.

## Abbreviations

RHR: Radial head resection
RHA: Radial head arthroplasty
ORIF: Open reduction and internal fixation
TEN: Titanium elastic nails
ROM: Range of motion
CT: Computed tomography
VAS: Visual Analog Scale score
ESAS: Elbow Self-Assessment score
MEPS: Mayo Elbow Performance score
DASH: Disabilities of the Arm, Shoulder, and Hand
PLRI: Posterolateral rotational instability
