# Correction to: a hybrid technique combining intramedullary pinning with extramedullary plate fixation in unstable and comminuted radial head fractures following on-table reconstruction

**DOI:** 10.1186/s12891-021-04552-7

**Published:** 2021-08-09

**Authors:** Xu Gao, Shi-you Dai, Hai-lei Yin, Fei Li, Yong-qiang Sui, Rui Huang, Hai-yu Fan

**Affiliations:** 1 Department of Orthopaedic Surgery, Qingdao University, Qingdao City, 266071 People’s Republic of China; 2 Department of Bone, Joint and Sports Medicine, East District, Qingdao Municipal Hospital, Qingdao City, 266071 People’s Republic of China; 3 Department of Second Orthopaedic Surgery, No. 971 Hospital of the People’s Liberation Army (PLA), Qingdao City, 266071 People’s Republic of China; 4 Department of State Key Laboratory for Marine Corrosion and Protection, Luoyang Ship Material Research Institute, Qingdao City, 266071 People’s Republic of China; 5 Department of Burn and Plastic Surgery, No. 971 Hospital of the People’s Liberation Army (PLA), Qingdao City, 266071 People’s Republic of China

**Correction to:** ***BMC Musculoskelet Disord*** **22, 613 (2021).**

**https://doi.org/10.1186/s12891-021-04498-w**

Following the publication of the original article [1] the authors noticed that the correction in Table 1 “the number of males is supposed to be 8 and the number of females is supposed to be 5” was not implemented. The original article [1] has been updated.

Below is the correct Table ﻿1.

**Table 1:** Details of patient demographics

	ORIF (experimental)	RHR (control)
Patients [13]	6	7
Male [8]	3	5
Female [5]	3	2
Mean ± SD age [years]	37.1 ± 9.0	41.7 ± 7.7
Side of the injury [13]	–	–
Right [9]	4	5
Left [4]	2	2
Mean ± SD time of follow-up [months]	38.6 ± 4.5	32.0 ± 6.3
Mode of injury [13]	–	–
Falling injury [4]	3	1
Traffic accident [9]	3	6
